# Underprepared: influences of U.S. medical students’ self-assessed confidence in immigrant and refugee health care

**DOI:** 10.1080/10872981.2022.2161117

**Published:** 2023-01-03

**Authors:** Shanna D. Stryker, Katharine Conway, Caitlin Kaeppler, Kelsey Porada, Reena P. Tam, Peter J. Holmberg, Charles Schubert, and Medical Student Global Health study group

**Affiliations:** a Department of Family and Community Medicine, University of Cincinnati College of Medicine, Cincinnati, Ohio, USA; b Department of Family Medicine, Boonshoft School of Medicine, Wright State University, Dayton, Ohio, USA; c Department of Pediatrics, Medical College of Wisconsin, Milwaukee, Wisconsin,USA; d Department of Pediatrics, University of Utah School of Medicine, Salt Lake City, Utah, USA; e Department of Pediatrics, Mayo Clinic, Rochester, MN, USA; f Department of Pediatrics, Cincinnati Children’s Hospital Medical Center, Cincinnati, OH, USA

**Keywords:** Refugee, immigrant, elective, medical student, undergraduate medical education, cultural competence

## Abstract

**Background:**

United States (U.S.) census data from 2017 indicates that the percentage of persons born outside of the U.S. is increasing. However, no studies describe the amount of class time focused on immigrant and refugee health during medical school in the U.S. nor on incoming residents’ confidence in providing culturally sensitive care. The objective of this study is to characterize final-year medical students’ exposure to immigrant and refugee health and their confidence in caring for these populations.

**Methods:**

A voluntary, cross-sectional survey was sent electronically to fourth-year medical students at twelve U.S. medical schools in 2020, with 707 respondents (46% response rate). Questions addressed respondents’ curricular exposure to immigrant and refugee health care during medical school and their confidence in providing culturally sensitive care. Chi-square tests were used to assess relationships between categorical variables, and odds ratios were calculated for dichotomized variables.

**Results:**

Most students (70.6%) described insufficient class time dedicated to culturally sensitive care, and many (64.5%) reported insufficient clinical exposure in caring for immigrants/refugees. The odds that incoming residents felt ‘usually’ or ‘always’ confident in their ability to provide culturally sensitive care to immigrants and refugees were higher in those with more class time on culturally sensitive care (OR 5.2 [3.6–7.4]), those with more clinical opportunities to care for immigrants and refugees (OR 7.2 [5.1–10.2]), and those who participated in a domestic low-resource or international elective (OR 1.4 [1.02–1.9]). More than half (55.3%) of respondents reported feeling ‘not at all’ or only ‘sometimes’ confident in their ability to provide culturally sensitive care to immigrants/refugees.

**Conclusions:**

Most fourth-year U.S. medical students entering residency feel unprepared to deliver culturally sensitive care to immigrants and refugees. This may be mediated by increased exposure to didactic curricula class time and/or experiential clinical activities, as those factors are associated with improved student confidence

## Introduction

Today’s medical students will enter practice caring for an increasingly diverse patient population. United States (U.S.) census projections show that between 2014–2060, the proportion of the population born outside of the U.S. will increase by 85% [1]. It is well-documented that patients with non-English language preference are at increased risk of suffering medical errors and worse clinical outcomes [2]. Provision of culturally sensitive care (care that offers services in a manner that is “relevant to patients’ needs and expectations” [3]) is associated with increased trust and satisfaction with medical care. Such patient care is also correlated with better treatment adherence and outcomes [4,5]. Studies of practicing physicians and residents describe concerns about their own preparedness to provide culturally sensitive health care [6–8]. Training health professionals to provide culturally sensitive care may increase clinician knowledge and attitudes [3], patient satisfaction [9], and patient involvement in their care [10]. Additionally, formal training and clinical activities on culturally sensitive care during residency can improve residents’ self-confidence to provide culturally sensitive care [11–13].

Dedicated global health (GH) curricula during residency not only provide opportunities to develop these skills but have also been shown to increase resident emphasis on the physical exam and resource-conscious practice, in addition to creating opportunities to learn to develop meaningful international partnerships [14–16]. Physicians who trained in residency programs with formal global health pathways describe feeling more prepared to care for immigrants, refugees, and patients with non-English language preferences [17]. Demand for training in GH and on immigrant and refugee health is high among residents [8,18,19]. GH opportunities in residency also generate interest from highly-ranked fourth-year medical student candidates [16,20,21]. However, barriers such as limited numbers of trained faculty, teaching time, and funding make providing these opportunities challenging [22].

In medical schools, there has been broad endorsement of cultural sensitivity training [23–25]. Additionally, there has been increasing integration of GH content for medical students as well as the development of competency-based guidelines for GH training [23–26]. In Europe and the United Kingdom, curricular reviews and surveys of faculty and students have shown increasing presence of GH content in undergraduate medical curricula [27–29]. Unfortunately, there is a lack of published work in the U.S. measuring graduating medical students’ perception of curricular time spent on immigrant and refugee health or students’ confidence in caring for these populations as they transition to the role of resident physicians.

To address this gap, members of the U.S. Midwest Consortium of Global Child Health Educators (MWC) developed a survey to answer the question ‘Do graduating U.S. medical students think that their curricula has prepared them to provide culturally sensitive care to immigrants and refugees?’. In addition, they sought to determine which learning opportunities positively impact graduating medical students’ self-assessment of their preparedness to provide culturally sensitive care to immigrant and refugee populations. The authors hypothesize that students do not feel adequately prepared to provide culturally sensitive care to immigrants and refugees at the time of graduation from medical school. The authors also hypothesize that students with more exposure to didactic and clinical time dedicated to the care of immigrants and refugees, and those who participate in GH experiences, feel better prepared to deliver culturally sensitive care. This paper will share results of the medical student survey developed by members of the MWC, the aim of which is to satisfy the first step of curriculum design: to provide a general needs assessment that helps to identify and describe a problem [30].

## Methods

To assess the feasibility of this study, an electronic pilot survey was emailed to fourth year medical students at the authors’ five institutions in March 2019. This pilot survey yielded 212 responses (a 32% response rate). Results showed that students perceive insufficient class time and clinical opportunities to care for immigrants and refugees. Based on feedback, adjustments were made to clarify that GH electives included domestic low-resource settings, and to improve survey distribution timing for better response rates. After presenting the results of the pilot survey to the MWC in fall 2019, members of seven additional institutions joined the study group.

The final cross-sectional survey was sent electronically through institutional listservs to fourth-year medical students at twelve U.S. medical schools in February 2020. Given the origin of this study with the MWC, most participating medical schools were in the Midwest region. Participation in the survey was voluntary, and participants could decline to answer any question or discontinue participation at any time. No identifying information was collected. Responses were collected using Qualtrics software. This study was approved or exempted by the IRB at each participating institution; some IRBs did not consider this study to be human subjects research due to the minimal risk and lack of collection of identifying information. After IRB approval or exemption, the study group sent an email containing an informational consent letter and the survey link to fourth-year medical student listservs at their institutions and arranged for two reminder emails to be sent. To reduce selection bias, a response rate of at least 25% from individual schools was required to be included in the data analysis; therefore, respondents from two schools were excluded.

Ultimately, 707 fourth-year medical students from ten different medical schools participated in this study, a 46% response rate based on medical school enrollment. This report is a sub-analysis of a survey that assessed medical student GH experiences and its impact on selecting residency training programs [31]. Here, results from the questions focused on immigrant and refugee care will be shared and discussed. The survey was developed based on the authors’ experiences as medical educators.

The explanatory variables measured were self-reported exposure to class time on immigrant and refugee health, self-reported frequency of opportunities to provide clinical care to immigrants and refugees, and participation in a GH elective during medical school. The outcome variable was self-reported confidence in their own ability to provide culturally sensitive care to immigrants and refugees. Any respondents who affirmed participation in a GH elective were asked to specify whether they had experience(s) with immigrants and refugees, at Indian Health Service (IHS) centers, the U.S.-Mexico border, in a local low-resource setting, and/or at an international site.

A four-point Likert scale was used to assess respondents’ reflections on the frequency of class time dedicated to the provision of culturally sensitive care for immigrants and refugees during medical school (no dedicated time, some dedicated time but not enough, adequate dedicated time, frequent dedicated time). The frequency of self-assessed opportunities to care for immigrants and refugees during clinical rotations was also evaluated using a four-point Likert scale (never, some but not enough, adequate encounters, frequent encounters). Lastly, students were presented with the statement ‘I feel confident in my ability to provide culturally sensitive care to immigrants and refugees’ and asked to choose one answer: not at all, sometimes, usually, or always.

Data were summarized using descriptive statistics. Chi-square tests were used to assess relationships between categorical variables, and odds ratios were calculated for variables dichotomized into ‘adequate’ vs. ‘inadequate’ or ‘more confident’ vs. ‘less confident’. A two-tailed alpha level of 0.05 was used to determine statistical significance.

## Results

Participants perceived a wide range of curricular content exposure and clinical opportunities related to the care of immigrants and refugees during their medical school training, as seen in Table 1. The majority reported insufficient class time (70.6% reported ‘no dedicated time’ or ‘some dedicated time but not enough’) and insufficient clinical opportunities (64.5% reported either no opportunities or ‘some but not enough’) to provide culturally sensitive care for immigrants and refugees. The majority (55.3%) of participants reported ‘never’ or only ‘sometimes’ feeling confident in their ability to provide culturally sensitive care to immigrants and refugees.

**Table 1.** Student responses to survey questions.

	n	%
**In medical school, I have had dedicated class time on how to provide culturally sensitive care to immigrants and refugees**		
No dedicated time	179	25.3
Some dedicated time but not enough	320	45.3
Adequate dedicated time	173	24.5
Frequent dedicated time	35	5.0
**In medical school, how often did you have the opportunity to provide clinical care to immigrants and refugees?**		
Never	74	10.5
Some, but not enough	382	54.0
Adequate encounters	200	28.3
Frequent encounters	51	7.2
**During medical school, did you participate in a Global Health elective rotation (e.g., internationally, local low-resource setting, at the US-Mexico border, with Indian Health Services, refugees, or immigrants)**		
Yes	220	31.1
No	487	68.9
**What type of Global Health elective did you participate in? (choose all that apply) *(n = 220)***		
International	168	76.4
Local low resource setting	101	45.9
Immigrant care elective	27	12.3
Refugee care elective	20	9.1
Indian Health Service	8	3.6
U.S.-Mexico border	4	1.8
Other	5	2.3
**I feel confident in my ability to provide culturally sensitive care to immigrants and refugees**		
Not at all	49	6.9
Sometimes	342	48.4
Usually	246	34.8
Always	70	9.9

**Figure 1.**
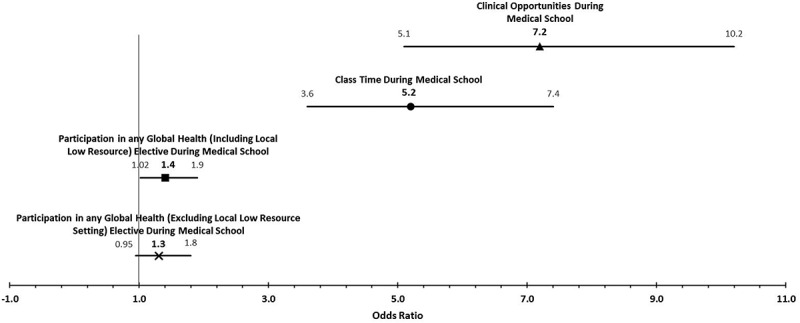
Odds of feeling confident to provide culturally sensitive care to immigrants/refugees.

Respondents who reported having ‘adequate’ or ‘frequent’ dedicated classroom instruction on providing culturally sensitive care to immigrants and refugees were more likely to ‘usually’ or ‘always’ feel confident in their ability to do so than those who reported insufficient class time on this topic (72.1% vs 33.3%; p < 0.001). As seen in Figure 1, the odds of feeling confident to provide culturally sensitive care were 5.3 times higher in those who reported having adequate or frequent class time dedicated to the care of immigrants and refugees, compared to those who reported ‘some dedicated time but not enough’, or no dedicated time (OR 5.2 [3.6–7.4]).

Those medical students who reported ‘frequent’ or ‘adequate’ opportunities to provide clinical care to immigrants and refugees during clinical rotations were more likely to report higher levels of confidence in their ability to provide culturally sensitive care to these populations compared to their peers who reported low exposure (74.1% vs 28.5%; p < 0.001). The odds of feeling confident to provide culturally sensitive care to immigrants and refugees was 7.2 times higher in those who reported frequent or adequate clinical encounters with immigrates or refugees, compared to those who reported inadequate or no clinical encounters (OR 7.2 [5.1–10.2]).

One-third (n = 220) of students participated in a GH elective. Of these, 86.8% (n = 191) specifically reported providing care in an international setting or to immigrant, refugee, or Native American populations within the U.S. Students who participated in any GH elective (whether it was in a local low-resource setting, an international setting, or a domestic rotation with immigrants and refugees, IHS, or at the U.S.-Mexico border) were more likely to report that they were ‘usually’ or ‘always’ confident in their ability to provide culturally sensitive care than students who had not had this experience (50.5% vs. 42.1%; p = 0.038). As seen in Figure 1, the odds of feeling confident to provide culturally sensitive care were 1.4 times higher in those who had participated in any GH elective compared to those who had not (OR 1.4 [1.02–1.9]), but not significant when those who worked in a local low-resource setting were excluded (OR 1.3 [0.95–1.8]).

## Discussion

This survey of graduating U.S. medical students was developed to describe their exposure to immigrant and refugee health curricula and preparedness to deliver culturally sensitive care. The survey also aims to describe the impact of didactic curricula, clinical opportunities, and GH electives on students’ preparedness to provide culturally sensitive care. The results show that most students perceived there to be insufficient class time and clinical opportunities focused on providing care to immigrant and refugee populations during medical school. In addition, a majority reported feeling ‘not at all’ or only ‘sometimes’ confident in their ability to provide culturally sensitive care to these populations, which supports the authors’ hypothesis. Secondary analyses suggest that a greater presence of focused didactics and clinical opportunities (local or international) during medical school is associated with higher confidence in providing culturally sensitive care. The findings affirm results of other studies which show that participation in GH elective opportunities improve clinicians’ confidence in their ability to provide culturally sensitive care, although only when all GH electives (including local low resource settings) were included, and with a low OR of 1.4 [6,32,33]. Clinical opportunities and didactic class time seem to have a greater impact on confidence than GH electives did. It is notable that just over half of those who participated in any GH elective rated themselves as ‘usually’ or ‘always’ confident in their ability to provide culturally sensitive care to immigrants and refugees. Follow-up questions asking details about these experiences or assessing the quality of these experiences were not asked, and this would be important for understanding why there is still a gap in the medical students’ confidence.

These results should provide medical schools and residency programs with the awareness of perceived gaps in knowledge and clinical confidence among graduating students. Employing multiple educational strategies and having well-trained and knowledgeable faculty to lead these efforts is important in preparing health professionals to care for a culturally diverse patient population [34]. More rigorous analysis and evaluation of teaching methods in this arena are imperative [25,35]. Some effective strategies can include opportunities for self-reflection [36], the use of virtual patients, partnering with interpretation services, paid community members as cultural mentors [37,38], peer education, and experiential learning opportunities [39].

Our study has limitations, including the use of a non-validated survey. In addition, the survey used self-assessments of confidence levels, and medical students’ perspectives of class time and clinical opportunities in lieu of directly assessing institutional curricula. Students’ assessments of adequacy of the amount of class time and clinical opportunities may also be related to their level of interest in this topic. Demographic information was not collected, and so demographic factors which may have been confounding variables were not able to be corrected for. Lastly, fixed-choice options were used; more in-depth qualitative information could have been obtained if the survey had allowed for free-text responses. Having an objective or patient-reported measure of students’ cultural sensitivity could provide resident educators with a more robust exploration of baseline preparedness; such scales have been validated in hospice workers and hospital-based providers [40].

## Conclusions

Most graduating U.S. medical students feel unprepared to deliver culturally sensitive care to immigrants and refugees. This may be mediated by increased exposure to didactic curricula and/or experiential clinical activities during medical school for all students, as those factors were found to be associated with improved student confidence in delivering culturally sensitive care. A critical next step in Kern’s approach to curricular development is a more targeted needs assessment measuring current exposure to immigrant and refugee health in didactic curricula and clinical rotations. Then, training best practices and ideal timing to develop the skillset of delivering culturally sensitive care can be studied and incorporated. Including immigrants and refugees in these investigations will be critical to affect quality of care metrics.
